# Editorial Notes

**Published:** 1949-01

**Authors:** 


### Editorial Notes

**Veterinary School Buildings.**

" And Samuel said, What meaneth then this bleating of the sheep in mine ears, and the lowing of the oxen which I hear ? " It is encouraging to observe that, a Professor of Veterinary Medicine having been appointed, a start has already been made with the buildings that the Department will require. We may begin to hope that at no very distant date it will be possible to restore the damage done to the Medical School and the Bristol General Hospital during the war.

**Medical Library.**

The new and revised edition of the Laws and By-Laws of the Bristol Medico-Chirurgical Society is being printed, and copies should be in the hands of members at an early date. To our other readers the chief interest lies in the concession made in a recent agreement with the University of Bristol : this extends to all subscribers to the *Journal* the privilege of borrowing books from the extensive medical library by post. The conditions are set out on a later page : this amenity applies only to individuals, not to institutions.

